# Immune Cells in Preeclampsia

**DOI:** 10.3390/ijms27010074

**Published:** 2025-12-21

**Authors:** Nathan Campbell, Marcus Robbins, Hellen Nembaware, Evangeline Deer, Denise Cornelius, Babbette LaMarca

**Affiliations:** 1 Department of Pharmacology & Toxicology, University of Mississippi Medical Center, Jackson, MS 39216, USA; ncampbell@umc.edu (N.C.); mkrobbins@umc.edu (M.R.); hnembaware@umc.edu (H.N.); edeer@umc.edu (E.D.); dcornelius@umc.edu (D.C.); 2 Department of Obstetrics & Gynecology, University of Mississippi Medical Center, Jackson, MS 39216, USA

**Keywords:** preeclampsia, immunology, autoimmunity, T cells, B cells, NK cells, macrophages

## Abstract

Preeclampsia (PE), new-onset hypertension during pregnancy, is associated with chronic inflammation both in the placenta and systemically. PE is characterized by placental ischemia, which then results in the production and release of anti-angiogenic factors and inflammatory mediators. Inflammation in PE leads to placental, renal, and vascular damage, which contribute to the phenotype of hypertension and organ dysfunction during pregnancy. T cells, B cells, Natural Killer cells, and macrophages have all been shown to play a role in the inflammation present in the disease. T helper cells contribute to the chronic inflammation in PE. They also activate B cells, which produce agonistic autoantibodies against the angiotensin II type 1 receptor. Natural Killer cells are activated in PE and shift away from decidual Natural killer cells, which produce angiogenic factors, and toward cytotoxic Natural Killer cells, which contribute to tissue damage. Macrophages are polarized towards proinflammatory subtypes and contribute to tissue damage and inflammatory signaling in PE patients. As the immune system plays a role in the pathophysiology of the disease, it may be a potential target for therapeutic intervention to improve maternal and fetal outcomes during and following a PE pregnancy.

## 1. Introduction

Preeclampsia (PE) is defined as new-onset hypertension after the 20th week of gestation alongside some form of organ dysfunction [1,2]. This organ dysfunction can manifest as proteinuria and kidney damage, cerebral effects and eclamptic seizures, liver dysfunction, abnormal blood clotting, and systemic endothelial dysfunction [1,2]. PE affects 5–7% of pregnancies in the United States every year and it is the leading cause of maternal and fetal morbidity and mortality during pregnancy [2]. The cause of PE is unknown but preexisting conditions such as hypertension, kidney disease, diabetes, or obesity can increase the risk for developing PE [3]. There is also a familial connection involved PE, as daughters born after a PE pregnancy are more likely to develop PE themselves [4]. Treatments for PE include antihypertensives to manage blood pressure and magnesium sulfate to prevent seizures but there is no cure for the disease beyond the delivery of the fetal–placental unit. PE is also recognized as a state of chronic inflammation and increased oxidative stress, both of which contribute to the pathophysiology of the disease [5,6,7]. It has been proposed that maternal infection may be an initiating factor that causes the initial inflammation leading to inflammatory activation. A systematic review revealed a two-fold increased risk for PE associated with bacterial and viral infections, though the exact mechanism is unclear [8]. Normal pregnancies cause adaptations in the thymus leading to changes in T cell development and resulting populations to improve immune tolerance throughout pregnancy [9]. Many different types of immune cells have been implicated in the inflammation during preeclampsia, including T cells, B cells, natural killer (NK) cells, macrophages, and others [5,10,11,12]. T helper cells are activated in PE patients and are shifted toward the proinflammatory Th1 and Th17 cell types [13,14]. Macrophages also shift towards the proinflammatory M1 cell type and away from the regulatory M2 cell type during PE [15]. B cells in PE patients produce an agonistic autoantibody against the angiotensin II type 1 receptor (AT1-AA), which binds to and activates the angiotensin II type 1 receptor (AT1R) [16,17,18]. The aim of this review is to discuss the differences in immune cell populations between normal pregnancies and preeclampsia.

## 2. Th1/Th2 Cells

CD4^+^ T helper (Th) cells are an important component of the immune system, and are responsible for directing adaptive immune responses, with T helper type 1 cells (Th1) and T helper type 2 cells (Th2) representing two key functional subsets [19]. Importantly, Th1 cells induce a cell-mediated immune response by differentiating in response to interleukin-12, resulting in a pro-inflammatory environment from the production of cytokines such as interferon-gamma (IFN-γ), tumor necrosis factor-alpha (TNF-α), and interleukin-2 (IL-2), which activate type 1 macrophages and support cytotoxic T lymphocyte responses [20,21]. In contrast, Th2 cells mediate humoral immune responses by differentiating in response to interleukin-4 (IL-4), resulting in the secretion of cytokines such as IL-4, IL-5, IL-10, and IL-13 to promote B cell maturation, IgE antibody production, and eosinophil activation [20,21]. These type 2 responses assist in the coordination of both humoral and eosinophil-mediated defenses against extracellular parasites from the activation of type 2 macrophages and the recruitment of eosinophils, basophils, and mast cells to an infection site [19]. However, during pregnancy, maintaining the functional balance between Th1 and Th2 cells is critical for immune regulation in order to protect both the mother and the developing fetus. Therefore, the balance between Th1 and Th2 immune responses is critical for sustaining maternal–fetal tolerance. Moreover, the relationship between these subsets is complementary because their cytokines can mutually inhibit each other’s differentiation, which assists in maintaining immune homeostasis.

### 2.1. Th1/Th2 Cells in Normal Pregnancy

During a healthy pregnancy, the maternal immune system undergoes dynamic changes to tolerate the semi-allogeneic fetus. Th1 cells are commonly upregulated in early pregnancy to support trophoblast invasion into the uterine spiral arteries, and Th1 cytokines become dominant during the peri-implantation period to assist in the trophoblast invasion rather than doing harm [22,23]. After placentation, there is a dynamic shift from Th1 to Th2 immunity occurring in the second and third trimesters, particularly at the maternal–fetal interface, to suppress Th1-mediated cytotoxic responses that could threaten fetal survival [22,23]. During this shift, Th2 cytokines facilitate the stimulation of other cells, such as cytotoxic T cells that have a vital role in promoting anti-inflammatory conditions, modulating immune cell function to support tissue remodeling, and protecting the fetus from harmful inflammation during pregnancy [23,24]. This new Th2-dominant environment facilitates trophoblast invasion, supports placental development, and helps establish an immune state that is essential for fetal growth, especially during the second trimester [23,24]. By maintaining this balance, Th2 cell-mediated responses aid in the prevention of pregnancy complications such as preeclampsia, miscarriage, and preterm labor that are associated with a shift back towards Th1 dominance and activation of NK cells and macrophages [24].

### 2.2. Th1/Th2 in Preeclampsia

Emerging evidence has demonstrated that a shift toward a Th1-dominant immune response contributes to systemic inflammation and placental dysfunction seen in preeclampsia. During normal pregnancy, a Th2 response supports immune tolerance; however, in PE, a shift toward Th1 polarization disrupts this balance, resulting in impaired placental function. Multiple studies have shown that a shift towards a Th1 dominance during pregnancy is responsible for the compromised placental function evident in preeclamptic women. In human studies, PE patients were shown to have a Th1 polarized shift and a reduction in Th2 cells in their peripheral blood profiles [25,26]. The increase in Th1 has also been linked with elevated concentrations of TNF-α and IFN-γ and lower expressions of transforming growth factor (TGF)-β1 and IL-10, which have also been associated with impaired trophoblast invasion, shallow placentation, and endothelial dysfunction compared with pregnant normotensive women [27,28]. Moreover, increased levels of Th1 pro-inflammatory cytokines have also been associated with other pregnancy complications such as intrauterine growth restriction (IUGR) and preterm birth [29,30]. Understanding the Th1/Th2 imbalance in preeclampsia not only elucidates its immunological underpinnings but may also offer potential biomarkers and therapeutic targets for early detection and intervention.

## 3. Regulatory T Cells

Regulatory T cells (Tregs) are a specialized subset of CD4^+^ T lymphocytes that arise primarily in the thymus (thymic Tregs, tTregs) during T cell development, although conventional CD4^+^ T cells can also differentiate into peripheral or induced Tregs (pTregs) in response to cytokines such as TGF-β in the periphery [31,32]. Tregs are defined by high expression of the CD25 and the transcription factor forkhead box P3 (FoxP3), which serves as a master regulator of their development and suppressive function [31,33,34]. Functionally, Tregs are central to maintaining immune homeostasis and self-tolerance, preventing autoimmunity and excessive inflammatory responses [32,33,35]. They suppress effector T cells and antigen-presenting cells through multiple mechanisms, including the secretion of inhibitory cytokines (IL-10, TGF-β, IL-35), metabolic disruption via IL-2 consumption, expression of CTLA-4 to downregulate costimulatory molecules on dendritic cells, and cytolysis of activated immune cells [36,37,38]. Beyond their role in immune suppression, Tregs are increasingly recognized for their tissue-specific specialization, adapting their phenotype to distinct microenvironments such as skin, gut, or reproductive tissues [39,40].

### 3.1. Regulatory T Cells in Normal Pregnancy

During pregnancy, Tregs are crucial for establishing maternal–fetal tolerance, allowing the semi-allogeneic fetus to develop despite continuous exposure to the maternal immune system [41,42]. The amount of Tregs increases rapidly at the maternal–fetal interface during early gestation, peaking in mid-pregnancy to support implantation and placental development [41,43]. Pregnancy-associated hormones, including progesterone, estrogen, and human chorionic gonadotropin, have been shown to promote Treg expansion and functional stability [44]. Within the decidua, Tregs interact with dendritic cells and trophoblasts to limit maternal effector T cell activation and promote an anti-inflammatory environment [45]. In addition to immunoregulation, Tregs contribute to early placental development by promoting trophoblast invasion and spiral artery remodeling [23,43,46,47]. Mechanistically, Tregs mediate tolerance by producing IL-10 and TGF-β, which suppress cytotoxic T cell responses, while also modulating NK cells’ activity and promoting angiogenesis [45,48,49]. The balance between Tregs and pro-inflammatory Th17 cells is especially important; an elevated Treg/Th17 ratio favors immune tolerance and successful pregnancy outcomes [50,51]. Experimental depletion of Tregs in mouse models leads to implantation failure and fetal resorption, further emphasizing their indispensable role [47,52]. In humans, reduced circulating or decidual Tregs have been linked to recurrent spontaneous abortion and other pregnancy complications [45,53,54].

### 3.2. Regulatory T Cells in Preeclampsia

Aberrations in Treg number and function are strongly implicated in the pathophysiology of preeclampsia [55,56]. Women with PE exhibit decreased proportions of FoxP3^+^ Tregs in both peripheral blood and decidual tissue, accompanied by reduced suppressive capacity [57]. This Treg deficiency is paralleled by an expansion of Th17 cells and other pro-inflammatory subsets, creating an imbalance that drives heightened immune activation [50,51,56,58]. Mechanistically, impaired Treg function contributes to defective trophoblast invasion, inadequate spiral artery remodeling, and endothelial injury through reduced IL-10/TGF-β production and increased pro-inflammatory cytokines such as IL-6 and TNF-α [59,60]. In vivo, the RUPP model has demonstrated that adoptive transfer of Tregs from normal pregnant rats improves hypertension and corrects the immune imbalance observed in PE [61]. Preclinical work suggests that therapeutic strategies aimed at boosting Treg activity, such as low-dose IL-2 administration, adoptive Treg transfer, may represent promising approaches to mitigate PE [56,61]. In addition, emerging strategies such as progesterone-mediated Treg induction and targeted stabilization of FoxP3 may offer further therapeutic benefit, although these approaches remain in early experimental stages [62,63,64].

## 4. Th17 Cells

IL-6 and TGF–β promote the differentiation of Th17 from naïve T cells [65]. T-helper 17 (Th17) cells, first identified in 2005, are a proinflammatory subset of CD4^+^ T cells that play important roles in autoimmune disorders, inflammation, and hypertension [66,67,68]. Since their discovery, Th17 cells have been linked to numerous inflammatory diseases, including rheumatic diseases, non-rheumatic autoimmune diseases, pulmonary diseases, atopic dermatitis, inflammatory bowel disease, and periodontal disease [66]. Recent studies have revealed that the transfer of Th17 cells to male mice results in characteristics of depression and learned helplessness, thus linking Th17 cells to the development of depression during pregnancy [69]. Th17 cells respond rapidly at inflammation sites, which serves as a link between the innate and adaptive immune cells [70]. They amplify neutrophilic responses, activate barrier epithelial cells, and stimulate antimicrobial peptide production [71]. Furthermore, Th17 cells primarily secrete IL-17 and IL-22. IL-17 notably enhances IL-6 production by the immune system [66,67,68]. While Th17 cells are critical for host defenses, their dysregulation has been linked to pregnancy-related morbidities such as preeclampsia, recurrent spontaneous abortion, and cholestasis of pregnancy. NK cells dampen inflammatory Th17 cells by secreting IFN-y to promote immune tolerance and successful pregnancy [72]. In vivo studies have shown that uncontrolled Th17 expansion can lead to fetal loss [72].

### 4.1. Th17 Cells in Normal Pregnancy

Pregnancy requires dynamic immune shifts, with pro-inflammatory responses in early pregnancy for implantation, anti-inflammatory states in mid-pregnancy for fetal growth, and pro-inflammatory again in late pregnancy for labor [73]. Tregs generally dominate over Th17 cells to prevent fetal rejection, though studies show inconsistent findings about whether circulating Th17 levels decrease, increase, or remain unchanged [51,74,75]. In normal pregnancy, Th17 cells secrete IL-17, which plays a vital role in angiogenesis and immune regulation. However, this role is not observed in the mouse placenta, suggesting that mouse models may not be reliable for investigating this function [76]. A balance between Th17 cells and regulatory T cells must be maintained because an excess in Th17 cell activity threatens the success of pregnancy [46]. Therefore, while dNK cells promote immune tolerance and vascular adaptation, Th17 cells provide immunity in a controlled way that complements the tolerogenic environment [13]. These factors collaborate to ensure pregnancy can proceed in a state of balance between resisting external pathogens and being permissive to the fetus [13,46,66].

### 4.2. Th17 Cells in Preeclampsia

**Figure 1:**
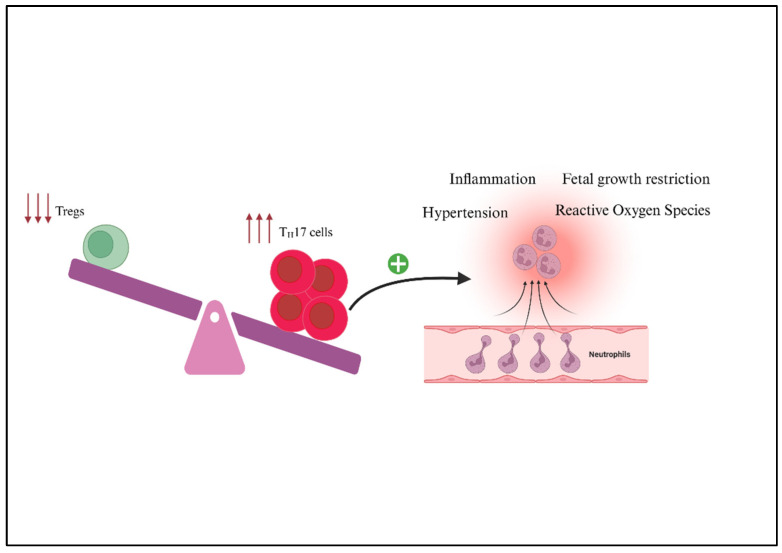
The imbalance between regulatory T cells (Tregs) and T helper-17 cells (Th17s) contributes to the pathogenesis of preeclampsia. In preeclampsia, Treg cells are reduced, and Th17 cells are increased, resulting in an elevated Th17:Treg ratio. This amplifies neutrophilic responses and shifts the immune environment to a pro-inflammatory state. As a result, there is increased inflammation, reactive oxygen species, fetal growth restriction, and hypertension.

Preeclampsia is strongly associated with abnormal trophoblast invasion and spiral artery remodeling [77]. Trophoblast invasion and vascular remodeling play a very important role during placentation; thus, inadequate remodeling results in decreased placental perfusion [78]. In addition, PE is associated with chronic inflammation and an imbalance among T-helper (Th) cell subtypes (Figure 1), particularly an increase in Th17 cells [61]. In vivo studies have shown that excess Th17 cells have been causing fetal loss [72]. Th17 cells are elevated in women with preeclampsia, and studies demonstrate that they synergize to impair placental function [67,79,80]. In studies done by Shields et al., the circulating Th17 population in normal pregnant rats was 1.16  ±  0.3%, which increased to 4.24  ±  0.8% after adoptive transfer of placental ischemia-stimulated Th17 cells [67]. This adoptive transfer significantly raised blood pressure from 96  ±  5 mmHg in NP rats to 118  ±  2 mmHg in NP+RUPP Th17 rats, while treatment with tempol, an antioxidant, lowered it to 102  ±  3 mmHg [67]. Furthermore, adoptive transfer of RUPP Th17 cells into NP rats significantly reduced pup weight (1.92 ± 0.09 g, *p* < 0.05) and placental weight (0.53 ± 0.01 g, *p* < 0.05), which is consistent with the IUGR observed in the RUPP control model of preeclampsia. Altogether, RUPP-induced Th17 cells play a direct role in mediating hypertension, inflammation, oxidative stress, and fetal growth restriction in preeclampsia [61,81].

## 5. B Cells

B cells are a population of lymphoid cells that express immunoglobulin (Ig) receptors and are critical for normal humoral immune function [82]. B cells are the antibody-producing cells of the immune system, and they contribute to immunological memory [83]. There are two distinct B cell populations, including innate-like B1 cells and classical B2 cells [82]. B1 cells arise from fetal progenitor cells and produce IgM antibodies that are polyreactive and bind to microbial antigens and self-antigens [84,85]. B1 cells produce these natural antibodies in the absence of antigen exposure and T cell help [86]. B2 cells are conventional B cells that undergo class switching and produce long-lasting, high-affinity IgG antibodies following T cell stimulation [87,88]. Mature B2 cells recognize their antigen through the B cell receptor (BCR) and internalize the antigen to present it via the MHC-II to a T cell receptor (TCR) on the surface of a T helper cell [89]. Upon T cell stimulation, B cells will transition into plasma cells, which are short-lived and produce large amounts of antibodies, or memory B cells, which are long-lived and provide long-term immune surveillance.

### 5.1. B Cells in Normal Pregnancy

**Figure 2:**
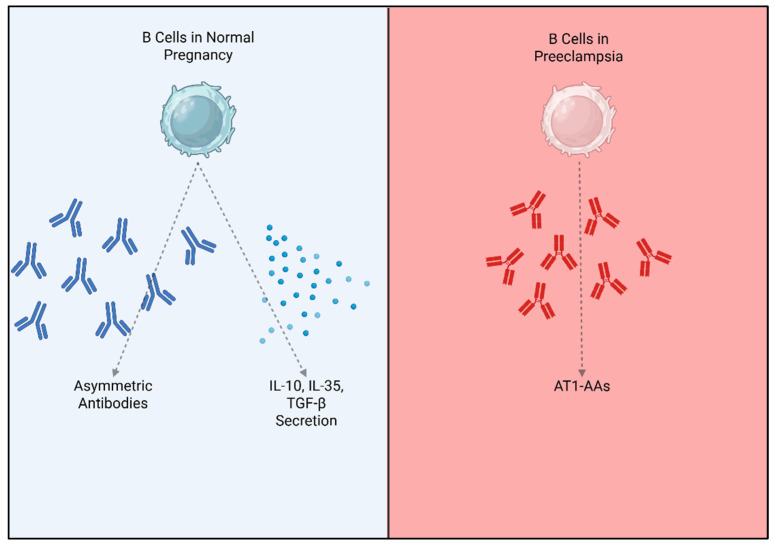
B Cells in normal pregnancy contribute to immune regulation through production of asymmetric antibodies and regulatory cytokines. In Preeclampsia B cells produce AT1-AAs contributing to the disease.

Pregnancy poses a significant challenge to the maternal immune system. Throughout pregnancy, maternal immune cells must create an environment of immune tolerance to support the semi-allogenic fetus. Although B cells only make up 1–2% of immune cells in the decidual, they are important in supporting the pregnancy [90]. During healthy pregnancies, B cells produce protective antibodies that inhibit the cytotoxic effects of other maternal lymphocytes [91,92]. There is some evidence that these protective antibodies come from the B2 cell population [93], though this is not fully clear. These protective antibodies are asymmetric with an oligosaccharide residue that prevents antibody complexes from forming or activation of natural killer cells [94,95]. B cell deficiencies, particularly in the memory and regulatory B cell populations, can increase the risk for preterm birth, recurrent pregnancy loss, and PE [96,97,98]. Additionally, B cells support healthy pregnancy through immune regulation from B regulatory cells that produce IL-10, inhibiting inflammation at the maternal–fetal interface (Figure 2) [99]. IL-10 can suppress T helper 1 and 17 cell differentiation and reduce the production of proinflammatory cytokines TNF-α and IFN-γ [100,101]. Some B cells also produce IL-35 during normal pregnancies, which also contributes to the regulation of T cell activation [102].

### 5.2. B Cells in Preeclampsia

Both B1 and B2 cells have been implicated in the pathophysiology of PE through the production of AT1-AA (Figure 2) [16,17,103]. A study by Jensen et al. in 2012 first implicated B1 cells in AT1-AA production in patients with PE [18]. They showed that B1a cell numbers and AT1-AA activity were correlated, and they found that B1 cells cultured with serum from PE patients produced AT1-AA in culture. Recent work from our group has also implicated B cells in the pathophysiology of PE, specifically the B2 cell population. We first showed that Anti-CD20 treatment improved PE symptoms in the RUPP rat model of PE [104]. We also showed that the communication between T cells and B cells via the CD40-CD40 ligand is critical for the development of a PE phenotype in rats [105]. Additionally, the adoptive transfer of B cells, specifically B2 cells from RUPP rats into NP rats, recaptures PE pathophysiology [106]. B cell Activating Factor (BAFF) is required for B2 cell stimulation, development, and maturation [107], and we have found that blockade of BAFF improves blood pressure and immune activation in RUPP rats [108]. Most recently, we have shown that human CD19+ B cells can cause a PE phenotype in pregnant rats associated with AT1-AA production and complement activation [109]. Other groups have recently shown that PD-1, an immune inhibitory signal, is reduced on immune cells, and specifically on B regulatory cells in PE patients [110,111]. The PD-1/PD-1L pathway is believed to be important for normal maternal immune tolerance during healthy pregnancies [112], and disruption of a key B cell subtype in PE may contribute to the overall chronic inflammation seen in the disease.

Recently, we demonstrated that offspring of RUPP rats continue to produce AT1-AA as adults, indicating a memory mechanism for its production [113,114]. This is supported by data showing that postpartum PE women can produce AT1-AA for up to 7 years following the PE pregnancy [115,116]. Interestingly, we found that depleting maternal CD20+ B cells with Rituximab, which improves maternal symptoms, also inhibited long-term production of AT1-AA in the offspring, thus further supporting the role of memory immune response in long-term consequences of PE for mother and child [114].

## 6. Macrophages

Monocytes migrate from the circulation into tissues, where they differentiate into macrophages [117]. Macrophages are key cells of the innate immune system and serve as an important part of the first line of immune defense [5,118]. Like all immune cells, macrophages originate from pluripotent hematopoietic stem cells in the bone marrow and are derived specifically via a myeloid progenitor [118,119,120]. In response to infection, inflammation, or tissue injury, they respond to chemotactic signals and migrate to the affected site [118,121]. At these sites, macrophages ingest pathogens and cellular debris through phagocytosis, digesting them in phagolysosomes formed by the fusion of phagosomes with lysosomes [118,121]. Importantly, the degraded products can be presented on major histocompatibility complex II (MHC II) molecules to activate antigen-specific immune responses involving T and B lymphocytes [118,119,120]. Beyond their role in phagocytosis and antigen presentation, macrophages can undergo polarization in response to microenvironmental stimuli [121,122,123,124]. Polarization generates distinct functional phenotypes, most notably classically activated (M1) macrophages and alternatively activated (M2) macrophages [122,125,126]. M1 macrophages are induced by IFN-γ and or LPS and characterized by the production of pro-inflammatory cytokines such as TNF-α, IL-1β, and IL-6. They typically express surface markers including CD80, CD86, TLR2, and iNOS [122,127,128]. In contrast, M2 macrophages are induced by IL-4, IL-13, or IL-10 and are associated with tissue repair and remodeling. M2 macrophages secrete anti-inflammatory cytokines such as IL-10 and TGF-β. Common surface markers that categorize macrophages as M2 are CD163, CD206, Fizz1, and Ym1/2. M2 macrophages are further divided into M2a, M2b, M2c, and M2d subcategories [122].

### 6.1. Macrophages in Normal Pregnancy

During pregnancy, decidual macrophages, which are maternal macrophages that reside in the decidua, contribute to spiral artery remodeling by producing angiogenic factors such as Vascular Endothelial Growth Factor (VEGF) and Placental Growth Factor (PlGF) [5,129]. They make up approximately 20–30% of decidual leukocytes in a healthy pregnancy [5,122]. In addition to decidual macrophages, Hofbauer cells, the fetal placental macrophages located within the chorionic villi, also play important roles in placental development and immune regulation [130,131,132,133].

Beyond their roles in placental development and immune regulation, macrophage polarization shifts dynamically across gestation. M1 macrophages predominate in the first trimester (<12 weeks), supporting embryo implantation, placental formation, and early development [5,15,134]. As placental formation and trophoblast invasion progress, both M1 and M2 macrophages are present [5,134]. After the placenta is fully developed in the second trimester, M2 macrophages predominate until labor, when a shift back toward M1 macrophages occurs. Maintaining this appropriate balance between M1 and M2 polarization has been shown to be essential for preserving immune tolerance and sustaining a healthy pregnancy after implantation [5,122].

### 6.2. Macrophages in Preeclampsia

**Figure 3:**
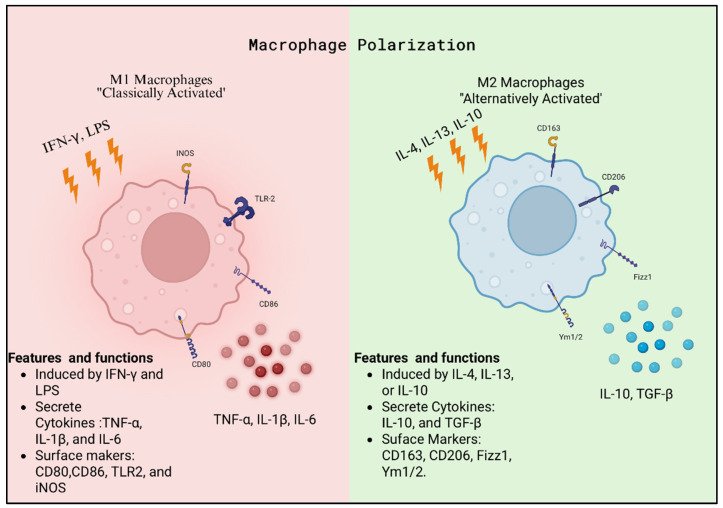
Macrophages are highly plastic innate immune cells with the ability to polarize their phenotype in response to the local microenvironment. M1 macrophages are classically activated macrophages induced by IFN-γ and LPS. They are characterized by surface markers such as iNOS, TLR2, CD68, and CD80, and they secrete pro-inflammatory cytokines including TNF-α, IL-1β, and IL-6. M2 macrophages, in contrast, are alternatively activated and are induced by IL-4, IL-13, or IL-10. They are defined by surface markers such as CD163, CD206, Fizz1, and Ym1/2, and they secrete anti-inflammatory cytokines, including IL-10 and TGF-β.

Disruptions in this carefully regulated balance of macrophage polarization have been implicated in pregnancy complications, such as preeclampsia, where altered macrophage function has been shown to play a role in the impairment of placentation and heightened inflammation (Figure 3) [5,122,127,128,135]. As mentioned earlier, in a healthy pregnancy, macrophages undergo a shift from M1 dominance in early gestation toward an M2 phenotype that supports trophoblast invasion and vascular remodeling [135]. In preeclampsia, this transition is impaired, with an increase of M1 macrophages and a reduction of M2 macrophages [15,136]. This imbalance constrains extravillous trophoblast invasion and spiral artery remodeling, partly through excessive secretion of inflammatory cytokines such as TNF-α and IL-1β and reduced production of the anti-inflammatory cytokine IL-10 [137,138]. Preclinical studies suggest that re-polarizing macrophages toward an M2 phenotype, through interventions such as mesenchymal stromal cell factors, statins, or cytokine modulation, may improve placental function and maternal outcomes [139,140,141,142].

## 7. Natural Killer Cells

NK cells are a type of white blood cell that play a significant role in the body’s line of defense. Unlike T and B cells, they do not require prior sensitization to recognize their targets [77]. Once activated, they release cytotoxic granules that contain perforin and granzymes, which induce apoptosis. They also secrete cytokines such as interferon-gamma and interferon-alpha to activate macrophages and dendritic cells to enhance the adaptive immune responses. Natural killer cells are found in various places, including the bone marrow, liver, uterus, spleen, and lungs. In humans, they are classified as CD3- CD56+ lymphocytes and can be distributed into two subtypes according to CD56 and CD16 expression. Uterine and decidual NK cells have higher CD56 density and less CD16 expression [143]. Additionally, they have larger granules compared to peripheral NK cells. Natural killer cells with larger granules are better cytokine producers, so this explains why the uNK cells are weakly cytolytic [144,145]. Uterine NK cells make up 50–70% of decidual lymphocytes in early pregnancy. They are essential for decidualization, a process that prepares the endometrium for embryo implantation by fostering placental development and modulating maternal immunity to prevent fetal rejection [146]. Decidual NK (dNK) cells are a subset of uterine natural killer (uNK) cells that differentiate in the decidua during early pregnancy. These two terms are often used interchangeably; however, uNK is a broader term that includes uterine NK cells across species, and dNK specifically refers to human decidual NK cells. They support placental development and spiral artery remodeling while maintaining reduced cytotoxicity to ensure maternal–fetal tolerance [147].

### 7.1. Natural Killer Cells in Normal Pregnancy

**Figure 4:**
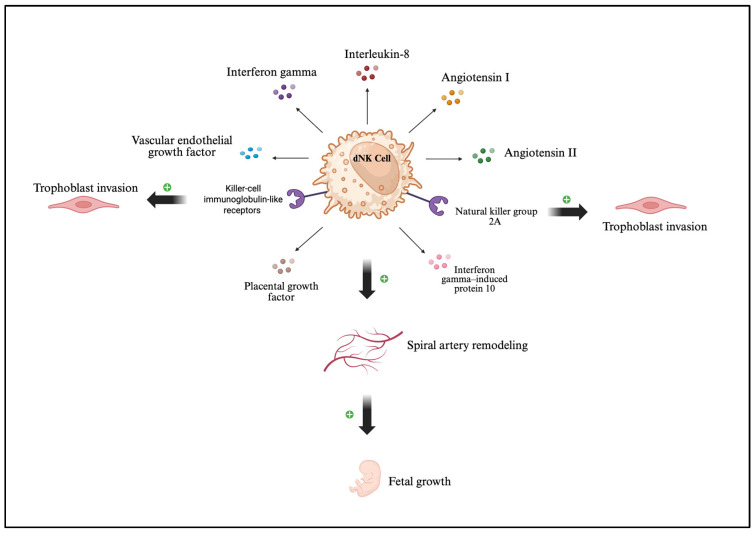
Decidual natural killer (dNK) cells secrete cytokines and growth factors such as interleukin (IL)-8, interferon (IFN)-γ, angiotensin I, angiotensin II, vascular endothelial growth factor (VEGF), placental growth factor (PlGF), and IFN-γ–induced protein 10 (IP-10). These factors collectively promote spiral artery remodeling and fetal growth. In addition, dNK cells express receptors such as killer-cell immunoglobulin-like receptors (KIRs) and natural killer group 2A (NKG2A) receptors, which interact with trophoblast cells to regulate trophoblast invasion.

In normal pregnancy, the maternal–fetal interface is composed of various immune cells, including dNK cells, macrophages, T cells, dendritic cells, B cells, and NKT cells [148]. It is where the mother’s uterine tissue (decidua) and the fetus’s placenta come into direct contact. This enables nutrient exchange, waste removal, and protection against infection. In early pregnancy, dNK cells have the highest expression level in the decidua [143]. These cells are distinct from peripheral NK cells in that they display low cytotoxicity and, conversely, rely on cytokine and growth factor secretion to achieve their functions [149]. Decidual NK cells are notable in their ability to interact with trophoblasts through inhibitory receptors such as KIRs and NKG2A to prevent immune reactions that may be harmful to the fetus [150]. In addition to this critical role, dNK cells secrete angiogenic factors including VEGF, PlGF, Ang I, and Ang II, which help remodel the spiral arteries in order to ensure adequate blood flow to the placenta (Figure 4) [149]. They also produce cytokines such as IFN-γ, IL-8, and IP-10, that carefully regulate trophoblast invasion as well as local inflammation in a way that supports the development of the placenta [149]. In studies done by Wang et al., uNK and dNK cells significantly promoted trophoblast proliferation through the production of IL-22, which has been shown to promote survival of human trophoblasts [151]. Moreover, coculture of uNK cells with the HTR8 cell line significantly promoted trophoblast invasion through the secretion of IL-8 and HGF [152]. Decidual NK cells directly contribute to fetal growth by releasing factors that promote the development of the embryo, underscoring the shift of NK cells from killer cells in the surrounding areas to regulators within the uterus [149].

### 7.2. Natural Killer Cells in Preeclampsia

The role of uNK and dNK cells in preeclampsia is complex. While some studies report that dNK cell numbers are significantly increased in preeclampsia, others find them decreased [153,154,155,156,157]. Thus, absolute cell counts may be less informative than examining the abnormal activation and functional changes of NK cells. In PE, peripheral and decidual NK cells undergo a type 1 shift, which reduces their ability to regulate spiral artery remodeling. This leads to abnormal placental vascularization and ischemia [150]. This polarization occurs independently of oxidative stress because treatment with tempol did not attenuate cNK activation in the placenta of NP rats receiving RUPP Th17 cells. Additionally, depletion of NK cells in RUPP rats significantly reduced the hypertensive response to placental ischemia. Therefore, RUPP-stimulated cNK cells directly increased blood pressure [150]. Al Omar et al. demonstrated that in vitro stimulation with IL-17 induced proliferation and increased perforin expression in human NK cells [158]. These findings suggest that there is an interplay between Th17 and NK cells; both the innate and the adaptive immune systems are involved in the development of the maternal systemic inflammation seen in women with preeclampsia [79]. Moreover, studies by Pazmany et al. revealed that patients with severe preeclampsia had reduced expression of Human Leukocyte Antigen-G (HLA-G) molecules [159]. These molecules, along with HLA-C and HLA-E, are expressed by extravillous trophoblasts and bound by NK cell receptors. Specifically, HLA-G protects trophoblasts from dNK cell lysis and is bound by NK cell receptor KIR2DL4 [160]. With HLA-G reduced, the semi-allogeneic fetus is left with limited protection against maternal immune responses, leading to impaired trophoblast invasion [161].

## 8. Sub-Characterizations of Preeclampsia

PE can be characterized as mild or severe based on the degree of the symptoms. Severe PE is diagnosed when blood pressure reaches >160/110 mmHg with signs of end-organ damage. PE is further divided into classifications of early onset or late onset based on diagnosis before or after 34 weeks of gestation [1,162]. Early onset PE carries a higher risk for maternal and fetal morbidity and mortality. Early onset PE can be considered a different disease as it is associated with a distinct genetic profile and changes in immune regulation [163]. Toll-Like Receptor 4 mutations have been identified in early-onset PE patients, and these patients also typically have higher levels of inflammatory biomarkers such as IL-6 and C-Reactive Protein [164]. This suggests that pro-inflammatory cells and signaling may contribute to the development of early onset PE as well as the increased severity in this form of the disease.

## 9. Clinical Context

Currently, the only “cure” for PE is the delivery of the fetal–placental unit. Treatment for PE focuses on managing symptoms with antihypertensives, such as labetalol, used to control blood pressure, and magnesium sulfate is used to prevent seizures [1]. Aspirin has also been investigated as a way to prevent PE in pregnancies that are considered higher risk for PE [165]. Targeting immune cells or mediators may be a viable strategy to improve symptoms and/or reduce the risks of PE. In animal models, immune targeting therapeutics such as etanercept, a TNF-α inhibitor; Rituximab, an anti-CD20 chemotherapeutic; ‘n7AAc’, an AT1-AA inhibiting peptide; and progesterone-induced blocking factor, a lymphocyte suppressor, all improve PE outcomes [81,114,166,167].

## 10. Conclusions

Immune cells play a critical role in normal pregnancy through promoting successful implantation and tolerance at the maternal–fetal interface. However, dysregulation of the immune system leading to chronic inflammatory activation is implicated in the pathophysiology of preeclampsia. T cells, B cells, Natural killer cells, macrophages, and other immune cells present in the maternal circulation and at the maternal decidua can all contribute to preeclampsia pathophysiology through the production of autoantibodies and cytokines, as well as causing tissue damage in the vasculature, kidney, and placenta.

## Data Availability

No new data were created or analyzed in this study. Data sharing is not applicable to this article.
